# Genome-wide association study on stem rust resistance in Kazakh spring barley lines

**DOI:** 10.1186/s12870-015-0686-z

**Published:** 2016-01-27

**Authors:** Yerlan Turuspekov, Danara Ormanbekova, Aralbek Rsaliev, Saule Abugalieva

**Affiliations:** Institute of Plant Biology and Biotechnology, Almaty, Kazakhstan; Research Institute for Biological Safety Problems, Gvardeiskiy vil, Dzhambul region, Kazakhstan

**Keywords:** Barley, Stem rust, Illumina array, Association mapping

## Abstract

**Background:**

Stem rust (SR) is one of the most economically devastating barley diseases in Kazakhstan, and in some years it causes up to 50 % of yield losses. Routine conventional breeding for resistance to stem rust is almost always in progress in all Kazakhstan breeding stations. However, molecular marker based approach towards new SR resistance genes identification and relevant marker-assisted selection had never been employed in Kazakhstan yet. In this study, as a preliminary step the GWAS (genome-wide association study) mapping was applied in attempt to identify reliable SNP markers and quantitative trait loci (QTL) associated with resistance to SR.

**Results:**

Barley collection of 92 commercial cultivars and promising lines was genotyped using a high-throughput single nucleotide polymorphism (9,000 SNP) Illumina iSelect array. 6,970 SNPs out of 9,000 total were polymorphic and scorable. 5,050 SNPs out of 6,970 passed filtering threshold and were used for association mapping (AM). All 92 accessions were phenotyped for resistance to SR by observing adult plants in artificially infected plots at the Research Institute for Biological Safety Problems in Dzhambul region of Kazakhstan. GLM analysis allowed the identification of 15 SNPs associated with the resistance at the heading time (HA) phase, and 2 SNPs at the seed’s milky-waxy maturity (SM) phase. However, after application of 5 % Bonferroni multiple test correction, only 2 SNPs at the HA stage on the same position of chromosome 6H can be claimed as reliable markers for SR resistance. The MLM analysis after the Bonferroni correction did not reveal any associations in this study, although distribution lines in the quantile-quantile (QQ) plot indicates that overcorrection in the test due to both Q and K matrices usage.

**Conclusions:**

Obtained data suggest that genome wide genotyping of 92 spring barley accessions from Kazakhstan with 9 K Illumina SNP array was highly efficient. Linkage disequilibrium based mapping approach allowed the identification of highly significant QTL for the SR resistance at the HA phase of growth on chromosome 6H. On the other hand, no significant QTL was detected at the SM phase, assuming that for a successful GWASmapping experiment a larger size population with more diverse genetic background should be tested. Obtained results provide additional information towards better understanding of SR resistance in barley.

**Electronic supplementary material:**

The online version of this article (doi:10.1186/s12870-015-0686-z) contains supplementary material, which is available to authorized users.

## Background

Barley is the second after wheat most important for Kazakhstan cereal crop with total annual grain yield of more than 2 mln tons [1]. SR caused by *Puccinia graminis* f. sp. *Tritici*, *Pgt,* is one of the most powerful factors affecting barley production in Kazakhstan. Sometimes annual yield losses exceed 50 % [2]. Currently, six genes are known to confer barley resistance to *Pgt* in the US, including well characterized *Rpg1* [3, 4], *rpg4* [5], and *Rpg5* [6, 7], and less studied *Rpg2, Rpg3,* and *rpg6* [8]. Most of these genetic studies were performed in relation to North American *Pgt* pathogen races MCCF and QCCJ by using conventional bi-parental genetic mapping approach. In addition, in response to serious threat of the new and highly infectious race of *Pgt* (TTKSK) in Africa and Near East, also known as Ug99, Steffenson with coauthors were able to identify qualitative *Pgt*-TTKSK locus on the long arm of chromosome 5H [9]. Also, Moscou with coauthors [10] showed that high-throughput genotyping in studies of bi-parental populations is a highly efficient tool for identification of novel QTLs associated with SR resistance to TTKSK. It was also concluded that hotspot on the chromosome 2H orchestrates largest inoculation-specific responses for enhanced resistance to the race TTKSK [10].

The other approach aimed for both known and novel agronomically important loci identification in cereals is based on AM, and high-throughput platforms with GWA scans [11]. In barley there are several reports demonstrating high efficiency of AM in genetic studies of quantitative morphological characters [12], abiotic stress tolerance [13], disease resistance [14], and grain quality [15]. In spite of some negative results [16, 17] the AM can be an appropriate approach for identification of novel genes controlling agronomically important traits, especially in new environments.

In Kazakhstan the genetic aspects of stem rust resistance were not studied well. Spring two-rowed barley represents over 90 % of all barleys in this country. Currently, despite regular use of conventional breeding procedures, there is no data regarding pathogen races classification in the region [2], and so far there were no attempts towards identification of valuable genes as well as their potential DNA markers for future marker assistant selection application in SR resistance local breeding programs. The main objective of this study was the genetic mapping of QTL associated with SR resistance based on AM for the local two-rowed spring barley lines in artificially infected field plots.

## Results

### Genetic variation in barley collection based on SNP markers

The 92 spring barley accessions from Kazakhstan (Additional file 1) were genotyped using the 9 K SNP iSelect array containing 7,842 SNPs. Genotyping revealed a set of 6,970 scorable SNPs (88.9 % of success) with 72.85 % variants being transitions and 27.15 % transversions. After quality control filtering of the SNP dataset 5,050 SNPs (64.4 % of success) were selected for further analysis. Chromosomal positions for each marker can be obtained from http://bioinf.hutton.ac.uk/iselect/app/index.pl of the James Hutton Institute, Scotland). The site provides information of chromosomal positions for 3,129 markers while remaining 1,921 SNPs were markers with unknown (U) positions. Additional information for each individual U marker can be retrieved from the Triticeae toolbox website (https://triticeaetoolbox.org). The total genetic length of the whole genome based on the genetic distances between mapped markers was 990.9 cM. The number of SNPs per chromosome with known positions ranged from 306 on chromosomes 1H and 4H to 608 on chromosome 5H, and suggested that average coverage for each marker was 0.33 cM.

**Fig. 1:**
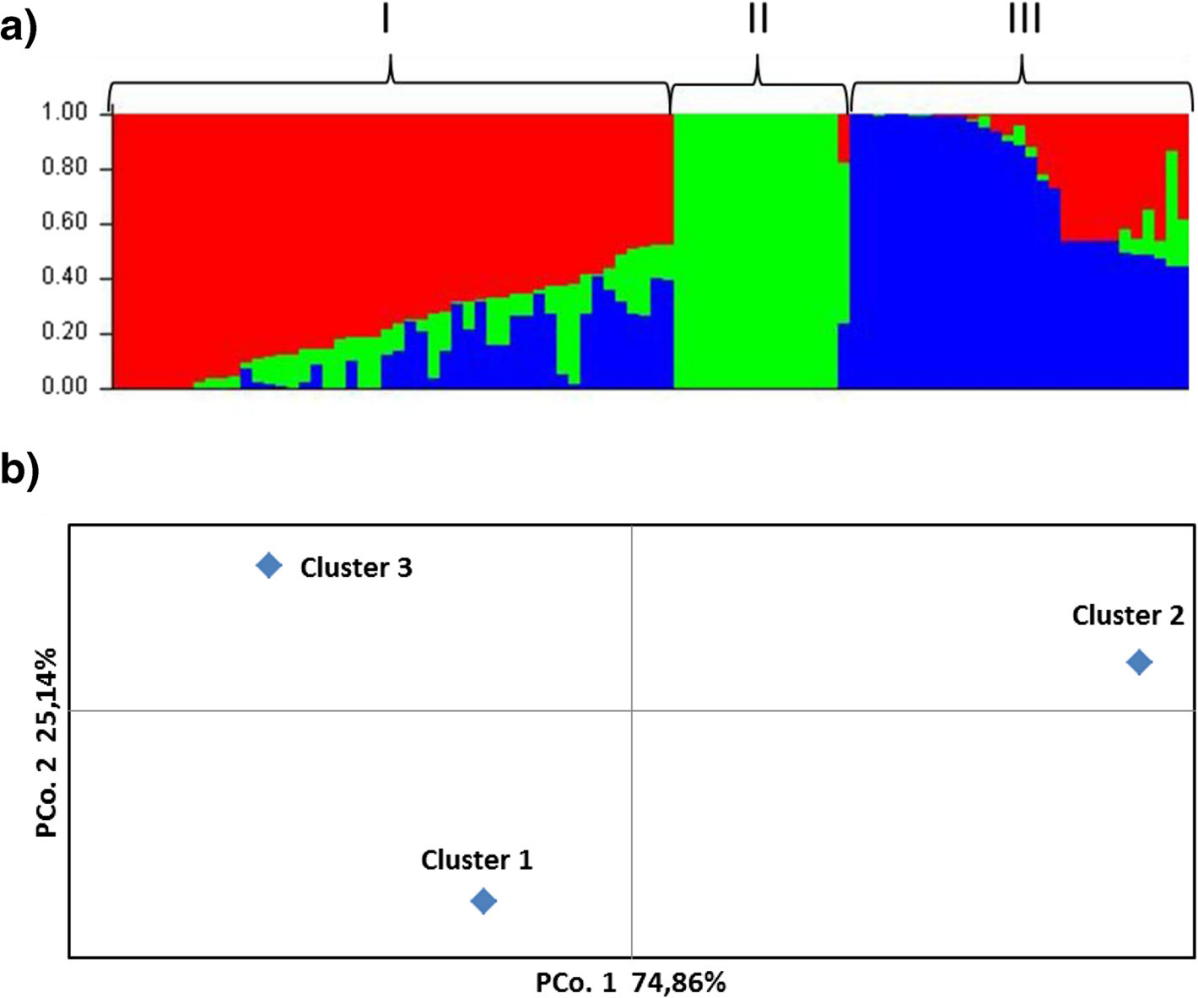
Subgrouping of 92 barley accessions from Kazakhstan based on 5050 SNP markers. **a** Clustering of accessions based on STRUCTURE software package and Delta K value (K = 3). Roman numbers are designate cluster order (see Table 1); **b** Principal Coordinate analysis of barley clustered groups using GenAlEx version 6.5

The subgrouping of the collection was studied using the STRUCTURE package [18] and the Delta K (ΔK) [19], indicating that the set of 92 selected barley lines consists of three clusters shown in Fig. 1. Genetic variation (Nei’s index) for Mean Total population was 0.26, with individual clusters ranging from 0.03 in cluster 2 to 0.41 in cluster 1 (Table 1). The majority of accessions in cluster I were the mixture of accessions collected from all six breeding stations, whereas in clusters II and III they were mostly collected in southern and central regions of Kazakhstan, respectively (Additional file 1). The level of the genetic variation is comparable with the one reported in our wheat study [20]. Analysis of molecular variances shows 73 % of variation within and 27 % among subgroups, suggesting that location difference is lower than the difference of plants within subgroups.

**Table 1:** Mean genetic diversity indexes in three barley subgroups based on 5050 SNPs^a^

Clusters:	I	II	III	Total
N^b^	48	15	29	92
I^c^	0,64	0,05	0,52	0,40
Uh^d^	0,41	0,03	0,35	0,26

^a^Single nucleotide polymorphism

^b^Number of accessions

^c^Shannon index

^d^Unbiased Nei’s diversity index

### Phenotypic evaluation and GWASmapping for QTL associated with stem rust resistance

Phenotypic evaluation of *Pgt* resistance was performed at the HA and SM phases. Despite the significant correlation (*P* < 0.05) between resistance at the HA and the SM phases, this result was in large discontent with allele status of *Rpg1* gene (Additional file 1). When alleles of *Rpg1* were converted to numeric figures and compared to resistance related observations at the HA and the SM phases no correlation were found. Thus suggesting that in Kazakhstan there are different pathogen races in comparison to the US. Overall, the majority of accessions (n = 85) showed high resistance to the *Pgt* at the HA, and 33 out of tested 92 accessions showed complete resistance to the pathogen population at the SM stage of growth (Additional file 1). The Shannon-Weiner genetic variation index of the sample in the SM (4.01) was significantly higher than in the HA (1.91) stage.

**Fig. 2:**
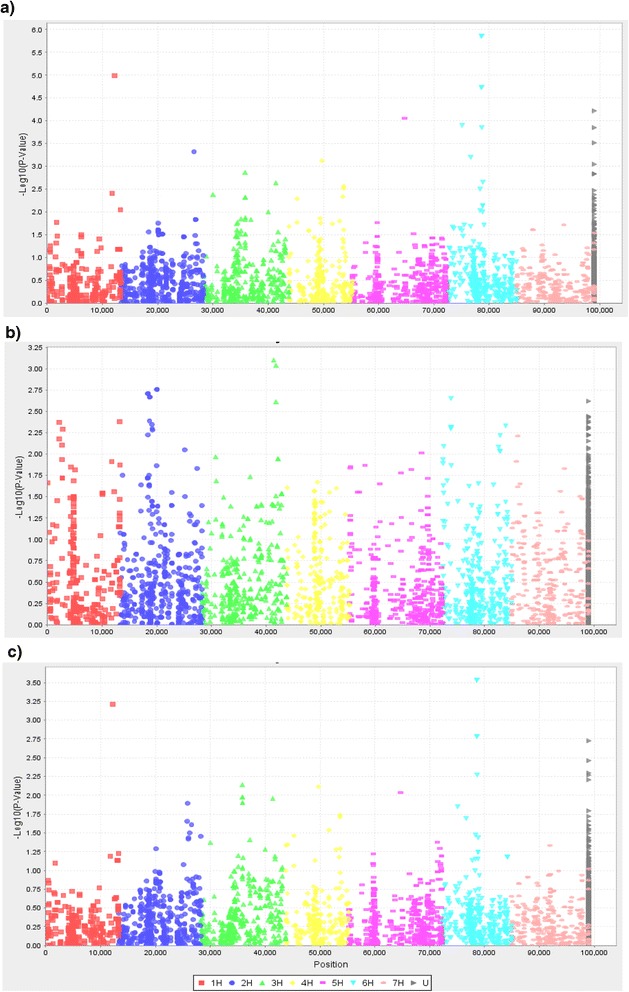
Manhattan plot of the AM study for barley *Pgt* resistance at the HA and SM phases using GLM and MLM tests. **a** GLM test at the HA growth phase; **b** GLM test at the SM phase; **c** MLM test at the HA phase

**Fig. 3:**
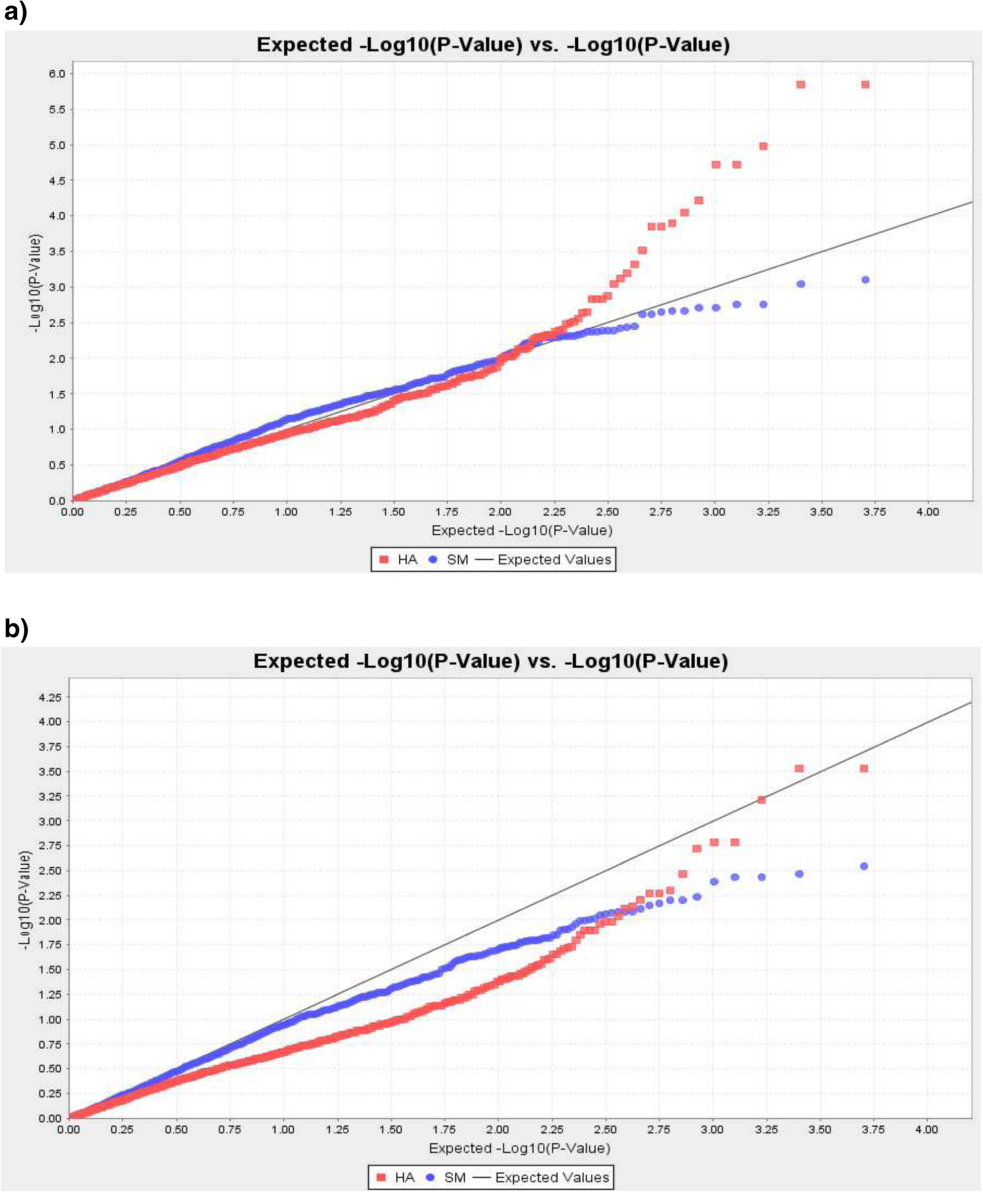
Quantile-quantile (QQ) plots of GWAS for *Pgt* resistance using GLM and MLM tests. **a** GLM-QQ plot using HA (red dots) and SM (blue dots) scores; **b** MLM-QQ plot, scans with and without Q matrix showed similar results

Data analysis by GLM allowed to detect fifteen SNPs at the HA stage, and two SNPs significantly associated with scores at the SM stage (Table 2, Fig. 2). However, after application of Bonferroni multiple test correction at 5 %, only two SNPs located on chromosome 6H were identified at HA phase. Though Shannon-Weiner genetic variation index of the population in the SM (4.01) was significantly higher than in the HA (1.91) stage whereas no any significant associations were found for scores in the SM stage. SNPs which were identified after Bonferroni correction application at the HA phase were genetically placed at the same position of the chromosome 6H (Table 2), indicating the presence of one significant QTL for *Pgt* resistance in the GLM experiment. Even less encouraging results were obtained using the MLM test. The MLM tests with and without use of the Q matrix at the HA stage showed identical results and allowed the identification of the same two candidate SNPs on the chromosome 6H (Fig. 2). However, no significant associations were found at the SM stage. Also, application of Bonferroni correction tests (*P* < 9.9036E-6) suggesting that there are no significant associations for *Pgt* resistance in the MLM experiment. On the other hand, it is interesting to note that QQ plots in the GLM test showed a higher correction efficiency of the population structure in comparison with MLM-QQ plot (Fig. 3). The distributions of *Pgt* resistance score in MLM-QQ plot were skewed to the right from the reference line, suggesting that overcorrection in the test due to use of Q and K matrices. Therefore, the distribution scores in the GLM-QQ plot provide reasonable support to the significance of QTL on chromosome 6H revealed by GLM test.

**Table 2:** Single nucleotide polymorphism (SNP) markers significantly (*P* < 0.001) associated with stem rust resistance at two growth phases (HA and SM) using general linear model (GLM

Marker	Phase	Chr.	cM	*P*	R^2^	Allele	MAF, %	Effect
SCRI_RS_216088	HA	1H	122,2	1.0365E-5	0.19524	T/C	C(0.12)	T(−0.95)
BOPA1_4235-1617	HA	2H	132,6	4.7965E-4	0.1287	G/A	G(0.23)	G(0.60)
SCRI_RS_96016	HA	4H	59,5	7.6236E-4	0.11891	A/C	C(0.14)	A(−0.69)
BOPA2_12_10844	HA	5H	93,9	8.9373E-5	0.15758	A/C	A(0.23)	A(0.66)
SCRI_RS_189878	HA	6H	28,5	1.2523E-4	0.15157	T/C	C(0.14)	T(−0.78)
BOPA1_5448-298	HA	6H	44,1	6.4042E-4	0.12209	A/G	G(0.18)	A(−0.63)
BOPA1_8005-914	HA	6H	63,5	1.8827E-5	0.18494	A/G	G(0.16)	A(−0.81)
BOPA2_12_30637	HA	6H	63,5	1.8827E-5	0.18494	A/G	G(0.16)	A(−0.81)
BOPA1_7370-818	HA	6H	63,5	1.4199E-6*	0.22878	G/A	A(0.14)	G(−0.96)
SCRI_RS_152841	HA	6H	63,5	1.4199E-6*	0.22878	A/C	C(0.14)	A(−0.96)
BOPA1_6205-683	HA	6H	63,9	1.4318E-4	0.15073	A/G	G(0.18)	A(−0.70)
BOPA1_2026-302	HA	U	0	5.9975E-5	0.16635	A/G	G(0.17)	A(−0.76)
BOPA1_5611-811	HA	U	0	9.0519E-4	0.11825	G/A	G(0.16)	G(0.65)
BOPA2_12_30158	HA	U	0	3.0675E-4	0.13688	A/G	A(0.20)	A(0.65)
BOPA1_5554-1971	HA	U	0	1.4318E-4	0.15073	A/G	G(0.18)	A(−0.70)
BOPA2_12_21386	SM	3H	131,3	7.9601E-4	0.11937	C/A	A(0.39)	C(−1.05)
SCRI_RS_189710	SM	3H	135,6	9.1218E-4	0.11685	A/G	G(0.32)	A(1.09)

*P* values indicated by star are significant after Bonferroni correction

## Discussion

Barley plants react to the stem rust pathogen invasions in different ways, ranging from the generation of toxic chemicals and programmed cell death [10, 32]. Depend on particular race of the pathogen the defense mechanism is also differentially triggered [5–8]. Therefore, studies of pathogen-host interactions in different environments expected to provide better evidence for understanding of *Pgt* resistance mechanism in barley.

In Kazakhstan, spring barley is growing on more than 1.5 million hectares annually and it is one of the most important export commodity to neighboring countries. Currently the average grain yield of spring barley in Kazakhstan is about 2.0 tons per hectare [1]. Such a low output can be explained by strong pressure from abiotic factors, such as drought, heat, and heavy rains in autumn, and by periodic invasions of devastating barley pathogens. Stem rust is among most harmful and widespread fungal diseases of barley in this country [2]. Planting of resistant barley varieties provide most sufficient and positive effect on management strategy for reducing yield losses due to stem rust. Conventional breeding for resistant lines is a very time-consuming and unpredictably challenging process. Therefore, molecular markers application to assist the identification and selection of stem rust resistance is a key for successful progress in corresponding breeding programs.

The barley 9 K Illumina SNP array [21], which is actively in use for various GWAS mapping projects [12–15], is one of those promising genomic tools for molecular markers identification and development. Here we report that genome wide genotyping of 92 spring barley accessions from Kazakhstan using the 9 K Illumina SNP array was highly efficient. Success rate of 88.9 % is comparable to the result in published report [22]. Genetic variability level was similar to the one obtained in our wheat genotyping study [20]. Notably, the clustering of studied population allowed separation of all accessions into three distinct subgroups (Table 2), where cluster II (n = 15) showed very low genetic variability level, which is most likely negatively affected our results in the GWAS.

In this study, the *Pgt* resistance was phenotypically evaluated for adult plants at the HA and SM stages. The resistance scores at the HA phase can be potentially important for detection of early activated pathogen-associated molecular patterns, which can trigger non-specific defense cascades. On the other hand, the resistance at late stages of growth, such as the SM, can be important for the identification of key resistance proteins [10, 23, 24]. It is interesting to note that despite significant correlation between two phenotypic scores (Pearson index, *P* < 0.05), the GWAS mapping scans showed different results (Fig. 2), supporting the hypothesis that different genes can be activated on various stages of growth. Whereas both GLM and MLM tests for the HA score allowed the identification of QTL on chromosomes 1H and 6H (Tables 2 and 3), none of these signals were significantly important at the SM stage (Table 3, Fig. 2). Vice versa, the identified minor QTL for the SM stage (GLM test) on chromosome 3H (Table 2) was not significant at the HM stage. Hence, the results underline importance to test resistant reactions of plants at different stages of plant growth [23, 24].

**Table 3:** Single nucleotide polymorphism (SNP) markers significantly (*P* < 0.001) associated with stem rust resistance at two growth phases (HA and SM) using mixed linear model (MLM)

Marker	Phase	Chr.	cM	*P*	R^2^	Allele	MAF, %	Effect
SCRI_RS_216088	HA	1H	122,2	6.1764E-4	0.13839	T/C	C(0.12)	C(1.09)
BOPA1_7370-818	HA	6H	63,5	2.9592E-4	0.15583	G/A	A(0.14)	A(1.18)
SCRI_RS_152841	HA	6H	63,5	2.9592E-4	0.15583	A/C	C(0.14)	A(−1.18)

In total, the GWAS allowed the identification of 17 candidate SNPs associated with SR resistance at two growth phases (Table 2). The use of 5 % Bonferroni correction showed that only one QTL on 6H is fitting to this test. However, functional genomics related information for each SNP marker (Table 2) provide some interesting results. For instance, at Triticeae toolbox (https://triticeaetoolbox.org) shown that SNP SCRI_RS_216088 on 1H is part of the NB-ARC domain which is shared by a number of R genes in plants [21]. Therefore, the potential significance of all identified SNPs as SR resistance markers must be re-evaluated in more comprehensive research involving wider germplasm resources, as well as bi-parental mapping populations.

In this study GLM and MLM tests predictably showed different outcome, as the MLM is a statistically more strict approach, and no significant QTL was detected in former model after application of the Bonferroni correction. On the other hand, the results of MLM-QQ plots clearly showed overcorrection in the test due to the use of K and Q matrices. Meanwhile GLM-QQ plot shows nearly perfect distribution of scores with slight deviation from the reference line in the region of 0.75-1.50 (Fig. 3a). This reveals the significance of GLM scans for the identification of candidate genes for such complex traits such as SR resistance. Therefore, in this analysis the evaluation of QQ plots suggests the importance of test of both general and mixed linear models in GWAS. The failure to identify any significant QTL using MLM scan is a clear indication on necessity to exploit larger sizes of studying population with more diverse genetic background.

## Conclusions

The resistance to SR is among most serious problems preventing barley grain yield increase in Kazakhstan. In this study we applied GWASmapping approach using 9 K SNP Illumina array for 92 local barley accessions and phenotypic scores for SR resistance at the different growth stages. The result shows that genome wide genotyping of spring barley accessions from Kazakhstan using the 9 K Illumina SNP array was highly efficient. The accessions were separated to three distinct subgroups and the level of the genetic diversity of total population was comparable to the results reported by Alqudah with co-authors [22]. The AM approach allowed the identification of two SNPs on chromosome 6H that related to *Pgt* resistance at the HA stage, but failed detecting any associations at the SM stage. Presumably, despite large set of SNP data, the studied 92 accessions is a too low number of samples for searching of a robust QTL of SR resistance in the particular environment. The genetic position of identified SNPs of the QTL at the HA stage did not coincide with chromosomal positions of known genes, such as *Rpg1*, *rpg4/Rpg5*, *Pgt*-TTKSK, and, therefore, they can be potentially new genetic factors related to SR resistance. Assuming that barley resistant mechanism to *Pgt* is triggered by pathogen races common in the Central Asia, particularly in Kazakhstan, the results add new insights in complex problem associated with resistance to SR.

## Methods

### Plant material

The collection of 92 two-rowed spring barley accessions was selected from six different breeding stations of Kazakhstan, and it is part of larger collection, which earlier studied in field trials in seven regions of the country [1]. The list includes 17 commercial cultivars officially registered at the Seed State Trial Commission of the Republic of Kazakhstan (Additional file 1).

### Stem rust resistance evaluation

Evaluation of resistance of barley genotypes was done in the Dzhambul region of Kazakhstan (43°31’N; 75°15E, elevation 743 m above sea level, average rainfall = 190 mm) at the Research Institute for Biological Safety Problems. Plants were grown in two randomized replication rows, with 30 cm distance between the rows. Plots were inoculated in the spring at the tillering stage with a mixture of isolates representing the most prevailing races of the pathogen in Central Asia. To analyze the accumulation of infection and high disease pressure, every five experimental plots were sown with universally susceptible varieties. Assessment of stem rust disease conducted at heading time and seed milky-wax maturity phases was done using modified Cobb Scale [25], and mean results over two replications shown in Additional file 1. The host response to the infection at the heading time was scored using “R” or resistant (small uredinia surrounded by chlorosis or necrosis); “MR” or moderately resistant (medium sized uredinia surrounded by chlorosis or necrosis); “MS” or moderately susceptible (medium large compatible uredinia without chlorosis and necrosis); and “S” or susceptible (large, compatible uredinia without chlorosis and necrosis). The mean SR resistance converted to numeric figures from 0 to 4 was used for GWAS mapping as a response factor.

### DNA genotyping and genetic variation study

DNA samples were extracted and purified from single seeds of individual cultivars using commercial kits (Qiagene, CA, USA). The DNA concentration for each sample was adjusted to 50 ng/ml. All samples were genotyped by the *Rpg1* gene according to [3, 4]. Also, accessions were genotyped using the barley 9,000 Illumina iSelect SNP array [26] at the Traitgenetics GmbH (Gatersleben, Germany). The Illumina Infinium procedure was performed according to the manufacturer’s protocol. SNP genotype analysis was carried out using the Illumina Genome Studio software (GS V2011.1). Population genetic analysis and principal coordinate analysis were performed using GenAlEx 6.5 [27, 28].

### Association mapping study

The SNP dataset was filtered using a 10 % cutoff for missing data and markers with minor allele frequency ≥ 0.10 were considered for GWAS. Numbers of hypothetical groups ranging from k = 1 to 10 were assessed using 50,000 burn-in iterations followed by 100,000 recorded Markov-Chain iterations. To estimate the sampling variance of population structure inference, five independent runs were carried out for each *k*. The output from STRUCTURE was analyzed for delta K value (ΔK) in STRUCTURE HARVESTER [19]. On the basis of the final *k* values, Q-matrix for three identified clusters was developed. GWASmapping of QTL governing stem rust response in the set of 92 accessions was performed using 5,050 informative SNPs, GLM and MLM tests [29, 30], and implemented in the TASSEL 5 package [31].

## Additional file

Additional file 1:
**List of studied cultivars and promising lines of spring barley growing in Kazakhstan.** (DOC 166 kb)

## Abbreviation

AM: Association mapping
GWAS: Genome wide association study
*Pgt*: *Puccinia graminis* f. sp. *Tritici*
HA: Heading time
SR: Stem rust
SM: Seed maturation
SNP: Single nucleotide polymorphism
K: Kinship
R: Resistant
MR: Moderately resistant
MS: Moderately susceptible
S: Susceptible
QTL: Quantitative trait loci
