# Supplementary material for: Genome-wide association study on stem rust resistance in Kazakh spring barley lines
Source: BMC Plant Biol. 2016 Jan 27;16(Suppl 1):6. doi: 10.1186/s12870-015-0686-z (PMC4895317; doi:10.1186/s12870-015-0686-z)
Supplement: Additional file 1: — List of studied cultivars and promising lines of spring barley growing in Kazakhstan. (DOC 166 kb) [file 12870_2015_686_MOESM1_ESM.doc]

Table 1. List of studied cultivars and promising lines of spring barley growing in Kazakhstan

| **ID** | **Cultivar/line name** | **Cultivar/prospective line status in Kazakhstan** | **Year of release** | **Region of growth in Kazakhstan** | **Breeding station** | **Cluster in Fig 1.**  **based on use of STRUCTURE** | **Average SR resistance score at HA phase** | **Average SR resistance score at SM phase** | **Allelic status of *Rpg1*** |
| --- | --- | --- | --- | --- | --- | --- | --- | --- | --- |
| **KZB01** | Nurinskiy 1 | prospective | n/a | Center | Karaganda | III | R | MR | R |
| **KZB02** | Medicum 11 | released | 1998 | Center | Karaganda | III | MR | MS | R |
| **KZB03** | Karagandinsky 2 | prospective | n/a | Center | Karaganda | III | R | MS | R |
| **KZB04** | Medicum 142 | prospective | n/a | Center | Karaganda | III | R | R | R |
| **KZB05** | Karagandinsky 7 | prospective | n/a | Center | Karaganda | III | R | MR | R |
| **KZB06** | Medicum 104 | prospective | n/a | Center | Karaganda | III | R | S | R |
| **KZB07** | Medicum 176 | prospective | n/a | Center | Karaganda | I | R | S | R |
| **KZB08** | Medicum 127 | prospective | n/a | Center | Karaganda | III | R | MR | R |
| **KZB09** | Medicum 156 | prospective | n/a | Center | Karaganda | III | R | MR | R |
| **KZB10** | Medicum 163 | prospective | n/a | Center | Karaganda | III | R | R | R |
| **KZB11** | Medicum101 | prospective | n/a | Center | Karaganda | III | R | MR | R |
| **KZB12** | Medicum 373 | prospective | n/a | Center | Karaganda | III | R | R | R |
| **KZB13** | Medicum 376 | prospective | n/a | Center | Karaganda | III | R | R | R |
| **KZB14** | Medicum 349 | prospective | n/a | Center | Karaganda | III | R | R | R |
| **KZB15** | Medicum 365 | prospective | n/a | Center | Karaganda | III | R | MS | R |
| **KZB16** | Medicum 318 | prospective | n/a | Center | Karaganda | III | R | MS | R |
| **KZB17** | Ubagan | released | 2003 | North | Karabalyk | III | R | MS | R |
| **KZB18** | Granal | released | 1991 | North | Karabalyk | I | R | S | R |
| **KZB19** | Karabalyksky 110 | released | 2010 | North | Karabalyk | I | MR | MS | R |
| **KZB20** | Druzhniy | released | 2005 | North | Karabalyk | I | MS | MR | R |
| **KZB21** | Medicum 85 | released | 1989 | North | Karabalyk | II | R | MS | R |
| **KZB22** | 19-89-01 | prospective | n/a | North | Karabalyk | III | R | MS | R |
| **KZB23** | 27-121-01 | prospective | n/a | North | Karabalyk | I | MR | MS | R |
| **KZB24** | 33-144-01 | prospective | n/a | North | Karabalyk | I | R | S | R |
| **KZB25** | Zhaik-2 | prospective | n/a | North | Karabalyk | I | R | R | R |
| **KZB26** | Tulpar | released | 2012 | North | Karabalyk | I | MR | MR | R |
| **KZB27** | Nutans 39 | prospective | n/a | North | Karabalyk | II | R | MR | S |
| **KZB28** | Ranniy | prospective | n/a | North | Karabalyk | III | R | R | S |
| **KZB29** | Pastbishniy | prospective | n/a | North | Karabalyk | II | R | MR | R |
| **KZB30** | Granal 447 | prospective | n/a | North | Karabalyk | I | R | R | R |
| **KZB31** | Atameken | prospective | n/a | South | Krasnovodopad | III | R | MS | R |
| **KZB32** | Baisheshek | released | 1985 | South | Krasnovodopad | III | S | S | R |
| **KZB33** | Bogara | prospective | n/a | South | Krasnovodopad | I | R | MS | S |
| **KZB34** | L-5/T-26 | prospective | n/a | South | Krasnovodopad | III | R | S | R |
| **KZB35** | Birlik | prospective | n/a | South | Krasnovodopad | II | R | S | R |
| **KZB36** | L-9/T-26 | prospective | n/a | South | Krasnovodopad | II | R | MR | R |
| **KZB37** | L-11/T-26 | prospective | n/a | South | Krasnovodopad | II | R | MR | R |
| **KZB38** | L-14/T-26 | prospective | n/a | South | Krasnovodopad | III | R | S | R |
| **KZB39** | L-217/T-26 | prospective | n/a | South | Krasnovodopad | II | R | R | S |
| **KZB40** | Krasnovodopad-100 | prospective | n/a | South | Krasnovodopad | II | R | S | S |
| **KZB41** | L-24/T-26 | prospective | n/a | South | Krasnovodopad | III | R | R | S |
| **KZB42** | L-28AG | prospective | n/a | South | Krasnovodopad | II | MR | S | S |
| **KZB43** | L-38/T-26 | prospective | n/a | South | Krasnovodopad | II | R | MS | S |
| **KZB44** | L-46/T-26 | prospective | n/a | South | Krasnovodopad | II | R | MS | S |
| **KZB45** | Asem | released | 2000 | Southeast | Almaty | I | R | MS | S |
| **KZB46** | Saule | released | 1991 | Southeast | Almaty | I | R | MS | S |
| **KZB47** | 2/84-6 | prospective | n/a | Southeast | Almaty | I | R | R | S |
| **KZB48** | 3/24-01 | prospective | n/a | Southeast | Almaty | I | R | R | S |
| **KZB49** | Syr Aruy | released | 2011 | Southeast | Almaty | I | R | S | S |
| **KZB50** | Susyn | released | 2008 | Southeast | Almaty | II | R | S | S |
| **KZB51** | Kymbat | prospective | n/a | Southeast | Almaty | I | R | MS | S |
| **KZB52** | 49/86-1 | prospective | n/a | Southeast | Almaty | I | R | MS | R |
| **KZB53** | 74/87-11 | prospective | n/a | Southeast | Almaty | II | R | R | R |
| **KZB54** | 76/86-241c | prospective | n/a | Southeast | Almaty | I | R | R | S |
| **KZB55** | Zhan | released | 2009 | Southeast | Almaty | I | R | R | S |
| **KZB56** | Inkar | prospective | n/a | Southeast | Almaty | I | R | MR | S |
| **KZB57** | 99/99-7 | prospective | n/a | Southeast | Almaty | I | R | R | S |
| **KZB58** | Elik | prospective | n/a | Southcenter | Kyzylorda | I | R | R | R |
| **KZB59** | 12/00-7 | prospective | n/a | Southcenter | Kyzylorda | I | R | R | R |
| **KZB60** | 103/99-11 | prospective | n/a | Southcenter | Kyzylorda | I | R | R | R |
| **KZB61** | 83/80-2 | prospective | n/a | Southcenter | Kyzylorda | I | R | MR | R |
| **KZB62** | Ular | prospective | n/a | Southcenter | Kyzylorda | I | R | S | S |
| **KZB63** | 41/99-7 | prospective | n/a | Southcenter | Kyzylorda | I | R | R | R |
| **KZB64** | 25/00-21 | prospective | n/a | Southcenter | Kyzylorda | I | R | R | R |
| **KZB65** | 59/87-30 | prospective | n/a | Southcenter | Kyzylorda | I | R | MS | R |
| **KZB66** | 46/00-14 | prospective | n/a | Southcenter | Kyzylorda | II | R | MR | S |
| **KZB67** | 74/99-4 | prospective | n/a | Southcenter | Kyzylorda | I | R | MR | S |
| **KZB68** | 1/99-3 | prospective | n/a | Southcenter | Kyzylorda | I | R | R | S |
| **KZB69** | 1/99-1 | prospective | n/a | Southcenter | Kyzylorda | I | R | R | S |
| **KZB70** | 53/86-20 | prospective | n/a | Southcenter | Kyzylorda | I | R | R | S |
| **KZB71** | 103/99-13 | prospective | n/a | Southcenter | Kyzylorda | I | R | R | S |
| **KZB72** | 122/99-6 | prospective | n/a | Southcenter | Kyzylorda | I | R | R | S |
| **KZB73** | 104/99-5 | prospective | n/a | Southcenter | Kyzylorda | II | R | MR | R |
| **KZB74** | 2/99-4 | prospective | n/a | Southcenter | Kyzylorda | I | R | MR | S |
| **KZB75** | 65/99-14 | prospective | n/a | Southcenter | Kyzylorda | I | R | MR | R |
| **KZB76** | Arna | released | 1997 | Southcenter | Kyzylorda | I | R | S | S |
| **KZB77** | ASHOS-163 | prospective | n/a | West | Aktobe | I | R | R | S |
| **KZB78** | ASHOS-164 | prospective | n/a | West | Aktobe | I | R | MR | S |
| **KZB79** | ASHOS-167 | prospective | n/a | West | Aktobe | III | R | MR | S |
| **KZB80** | ASHOS-168 | prospective | n/a | West | Aktobe | III | R | MS | S |
| **KZB81** | ASHOS-169 | prospective | n/a | West | Aktobe | I | R | R | R |
| **KZB82** | ASHOS-175 | prospective | n/a | West | Aktobe | III | R | S | S |
| **KZB83** | ASHOS-181 | prospective | n/a | West | Aktobe | I | R | MR | S |
| **KZB84** | ASHOS-182 | prospective | n/a | West | Aktobe | I | R | MS | S |
| **KZB85** | ASHOS-183 | prospective | n/a | West | Aktobe | I | R | R | S |
| **KZB86** | ASHOS-184 | prospective | n/a | West | Aktobe | I | R | MR | S |
| **KZB87** | ASHOS-185 | prospective | n/a | West | Aktobe | III | R | R | R |
| **KZB88** | ASHOS-187 | prospective | n/a | West | Aktobe | I | R | R | S |
| **KZB89** | Ilek-9 | released | 2007 | West | Aktobe | III | R | S | S |
| **KZB90** | Ilek-34 | released | 2006 | West | Aktobe | I | R | MR | R |
| **KZB91** | ASHOS-194 | prospective | n/a | West | Aktobe | III | R | R | R |
| **KZB92** | Yassy | released | 2000 | West | Aktobe | I | R | R | R |

^a^, information is not available
